# Interpretation of NMR Relaxation as a Tool for Characterising the Adsorption Strength of Liquids inside Porous Materials

**DOI:** 10.1002/chem.201403139

**Published:** 2014-08-21

**Authors:** Carmine D'Agostino, Jonathan Mitchell, Michael D Mantle, Lynn F Gladden

**Affiliations:** [a] Department of Chemical Engineering & Biotechnology, University of Cambridge Pembroke Street, Cambridge CB2 3RA (UK) E-mail: mdm20@cam.ac.uk

**Keywords:** catalysis, mesoporous materials, NMR spectroscopy, porous media, relaxation times, surfaces and interfaces

## Abstract

Nuclear magnetic resonance (NMR) relaxation times are shown to provide a unique probe of adsorbate–adsorbent interactions in liquid-saturated porous materials. A short theoretical analysis is presented, which shows that the ratio of the longitudinal to transverse relaxation times (*T*_1_/*T*_2_) is related to an adsorbate–adsorbent interaction energy, and we introduce a quantitative metric *e*_surf_ (based on the relaxation time ratio) characterising the strength of this surface interaction. We then consider the interaction of water with a range of oxide surfaces (TiO_2_ anatase, TiO_2_ rutile, γ-Al_2_O_3_, SiO_2_, θ-Al_2_O_3_ and ZrO_2_) and show that *e*_surf_ correlates with the strongest adsorption sites present, as determined by temperature programmed desorption (TPD). Thus we demonstrate that NMR relaxation measurements have a direct physical interpretation in terms of the characterisation of activation energy of desorption from the surface. Further, for a series of chemically similar solid materials, in this case a range of oxide materials, for which at least two calibration values are obtainable by TPD, the *e*_surf_ parameter yields a direct estimate of the maximum activation energy of desorption from the surface. The results suggest that *T*_1_/*T*_2_ measurements may become a useful addition to the methods available to characterise liquid-phase adsorption in porous materials. The particular motivation for this work is to characterise adsorbate–surface interactions in liquid-phase catalysis.

## Introduction

Surface interactions of liquids in porous media are of great importance, particularly in the field of heterogeneous catalysis,[1–5] and the ability to understand surface interactions is essential for efficient and rational catalyst design.[6] However, probing liquid–surface interactions in liquid-saturated porous media is particularly challenging. Established techniques for probing the interaction of molecules at solid surfaces include isosteric heat of adsorption, temperature-programmed desorption (TPD), infra-red (IR) spectroscopy, and nuclear magnetic resonance (NMR) chemical shift (*δ*). However, all these measurements have limitations, and none are able to probe, non-destructively, the behaviour of molecules on catalyst surfaces at realistic reaction conditions. For example, whilst isosteric heat of adsorption measurements are non-invasive,[7] they require the determination of adsorption isotherms at different temperatures, and are therefore extremely time-consuming; characterisation of co-adsorbed systems may take in excess of well over ten hours.[8] TPD is known to cause in situ reactions of some organic molecules leading to dehydration, isomerisation and decomposition, and is also limited in its ability to probe co-adsorption.[9, 10] IR is well known to be limited to liquids and solids that are transparent in the frequency range of interest.[11]

Finally, NMR chemical shift interpretation is robust only for monolayer surface coverage.[12, 13] It has recently been reported[14] that under certain conditions chemical shift measurements can be used to probe surface interactions over PVP-stabilised metal nanoparticles. In this example, the chemical shift of formic acid (adsorbate) on metal colloid catalysts, measured by ^13^C NMR spectroscopy in aqueous suspension, was used to infer the strength of surface interaction. However, to achieve this measurement it was necessary to avoid direct contact between the ^13^C atom of the adsorbate and the metal surface by inserting oxygen-atom spacers, in order to eliminate spectral line broadening. Whilst this is clearly an elegant measurement, it cannot readily be transferred to measurements of liquid–surface interactions in saturated porous materials of industrial relevance. In general, the use of NMR methods to probe adsorption is made challenging because of the broadening of the NMR lineshape associated with the adsorbed species, which arises because of the magnetic susceptibility differences between adsorbate and adsorbent. There exist many reports of NMR experiments probing different aspects of adsorption. ^13^C NMR is often the method of choice because of the wider chemical shift range, and hence spectral resolution attainable, relative to ^1^H observation. Examples of ^13^C NMR to study adsorption include chemical shift studies to investigate the structure of the adsorbed species[15] and the determination of acid site strength (mostly in zeolites) using suitable probe molecules such as acetonitrile or acetone.[16, 17] ^13^C NMR approaches usually require the use of enriched ^13^C species[15] due to the low signal-to-noise ratio of ^13^C signal at natural abundance. However, it is noted that ^13^C studies of adsorbed species at natural isotopic abundance using the distortionless enhancement by polarisation transfer (DEPT) technique have been reported,[3] although the strength of interaction was not probed using this technique. ^1^H NMR has been used by various researchers to study acidic strength of hydroxyl groups in zeolites, using deuterated probe molecules such as acetonitrile and pyridine.[17–19] For example, Zheng et al.[19] have conducted experimental and theoretical studies of deuterated pyridine adsorption to investigate acidic strength of solid acids and found a linear correlation between the ^1^H chemical shift of adsorbed pyridine and the proton affinity. Thus, whilst various implementations of NMR spectroscopy have been used to study adsorption phenomena in porous media, a robust, generic technique readily applicable to co-adsorption and with the ability to study liquid-saturated pore spaces, as opposed to monolayer coverages, has not been presented. The method proposed in the present work exploits the modification of the nuclear spin relaxation time characteristics of the adsorbate that result from its interaction with the surface of the pore. The advantage of using so-called NMR relaxation measurements is that the characterisation of the adsorption interaction does not rely on the NMR lineshape and the ‘peak position’—the actual peak position associated with liquid confined within a porous medium, or chemical shift, may be influenced by factors other than the adsorbate–adsorbent interaction alone.[20] Moreover, measurements based on chemical shift will be limited in terms of the systems that can be studied when multi-component liquid systems inside the pore space are of interest, because of the broad, overlapping, line shapes associated with each component. In contrast, the relaxation times of different chemical species in a multi-component system are often quite distinct even when their respective chemical shifts overlap. It follows that relaxation is a valuable probe of adsorbate–adsorbent interaction, particularly for liquid–surface interactions. Thus far[21–24] it has been used as a qualitative probe yielding the relative strength of interactions between different adsorbate–adsorbent systems. It is the purpose of this paper to demonstrate that the ratio of the spin-lattice to spin-spin (or transverse) relaxation time (*T*_1_/*T*_2_) can be related directly to the activation energy of desorption characterising the strongest adsorption sites on the surface of the adsorbent, as determined by temperature programmed desorption (TPD).

In recent years, NMR relaxation has emerged as a non-invasive, chemically sensitive technique for studying surface interactions of liquids in saturated porous media.[24] Following radio-frequency (rf) excitation, longitudinal *T*_1_ relaxation processes drive the longitudinal magnetisation to the equilibrium position, that is, aligned with the external magnetic field, whilst transverse *T*_2_ relaxation processes determine the rate of loss of phase coherence of the magnetisation in the transverse plane.[25] Reduced *T*_1_ and *T*_2_ relaxation times are observed when liquid molecules adsorb on a solid surface due to a change in the molecular mobility;[26] in bulk liquids, *T*_1_≈*T*_2_. Both *T*_1_ and *T*_2_ are affected by changes in the rotational correlation time of the adsorbate molecules. However, *T*_2_ is further influenced by a translational correlation time associated with surface diffusion.[27, 28] Consequently, when molecules adsorb on surfaces, changes in their translational and rotational dynamics influence *T*_2_ more than *T*_1_, resulting in *T*_1_>*T*_2_.[29] In porous materials with a high surface-to-volume ratio, *S*/*V*, the observed relaxation rates are proportional to *S*/*V*,[30] so absolute *T*_1_ and *T*_2_ measurements cannot be readily used to compare interactions between materials with differing pore geometry, pore size and density of adsorption sites. However, the ratio of relaxation times *T*_1_/*T*_2_ is (to leading order) independent of these characteristics. It has been observed empirically that the ratio *T*_1_/*T*_2_ provides an indication of the relative strength of surface interaction for different liquids in the same catalyst.[24] Therefore, *T*_1_/*T*_2_ provides a novel and robust approach for studying competitive adsorption processes. For example, in the aforementioned work,[24] it was observed that water imbibed in Pd/Al_2_O_3_ catalyst trilobes exhibits a much larger *T*_1_/*T*_2_ ratio (stronger surface interaction) than 2-butanone on the same surface; water is known to poison this palladium catalyst in the hydrogenation of 2-butanone by preferential adsorption on the active sites. Other studies, which exploit *T*_1_/*T*_2_ as a probe of surface interaction strength and the ability of these measurements to help understand catalytic performance, have recently been reported.[21–23] It is important to note that besides the advantage of being independent of pore geometry, the use of *T*_1_ and *T*_2_ relaxation times is particularly advantageous if co-adsorption in porous materials is to be probed. This is because the method has the advantage that different species can often be separated based on their relaxation time values even when the two species cannot be separated in the chemical shift domain, which is often the case in porous materials. This was shown when studying co-adsorption of various binary mixtures in porous Al_2_O_3_ and SiO_2_ supported catalysts.[24]

The aim of the present work is to confirm theoretically that the ratio *T*_1_/*T*_2_ can be used as a qualitative descriptor of surface affinity, and extend the measurement to provide a quantitative metric of the same. Moreover, if this is the case, can the *T*_1_/*T*_2_ measurement be shown to correlate with an accepted laboratory characterisation of the adsorption interaction, namely TPD? Indeed, if the theoretical interpretation summarised below is appropriate, we might expect the value of *T*_1_/*T*_2_ to correlate with the strongest adsorption energies characterising a particular system, since nuclear spin relaxation is dominated by the strongest relaxation sinks present.[31]

### Surface relaxation and adsorption

In this section we establish proportionality between NMR relaxation and adsorption energy for a molecule interacting with the surface of a pore; the relaxation of the adsorbed molecule will be modulated by the reduction in molecular mobility and the dipole–dipole coupling between protons in the adsorbed molecule and those bound to the surface.[26] The present analysis develops from the established theory of surface relaxation, which assumes the pore surface contains paramagnetic impurities that act as relaxation sinks and it is dipole-electron coupling that determines *T*_1_/*T*_2_. In recent work[27] we demonstrated that the analysis of Godefroy et al.[32] for proton–electron dipole coupling is extensible to proton–proton dipole coupling, albeit with a much weaker coupling constant.[33] In the absence of paramagnetic species, it is the active binding sites on the pore surface that are considered to be the relaxation sinks.

Molecules adsorbed onto a surface undergo two-dimensional (2D) translational motion governed by the activation energy *E*_m_. The diffusion coefficient associated with this surface motion is:


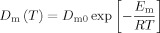
(1)

in which *D*_m0_ is a temperature independent contribution to surface diffusion, *R* is the ideal gas constant, and *T* is the temperature. In liquid-saturated porous materials, adsorbed molecules exchange with molecules not directly interacting with the pore surface. Such molecules will have motional characteristics very similar to those of the bulk liquid. The potential binding energy of adsorbed molecules governing the desorption rate is *E*_s_. The effective surface diffusion coefficient *D*_eff_ (observed) is modified from *D*_m_ due to the finite residence time on the surface. Therefore, the effective diffusion coefficient is:


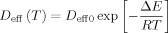
(2)

in which Δ*E*=*E*_m_−*E*_s_ is an activation energy for surface diffusion. The diffusion coefficient has an associated correlation time *τ*_m_ that describes the time for diffusion between active binding sites, such that *D*_eff_(*T*)≈*ε*^2^/(4*τ*_m_), where *ε* is the thickness of the adsorbed surface layer. Thus, the surface correlation time is defined as:


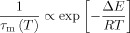
(3)

There will also be a surface residence time for adsorbed molecules, which is similarly defined as:


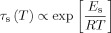
(4)

The observed relaxation times for liquid saturated porous media are:[34]


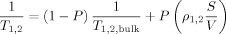
(5)

in which *ρ*_1,2_ are the surface relaxivity values governing longitudinal and transverse relaxation processes at the pore surface, respectively, *T*_1,2,bulk_ are the respective relaxation times of the bulk liquid, and *P* is the fraction of spins on the pore surface. For the catalyst supports of interest, the pore diameters are on the order of nanometres, such that the observed relaxation times are determined only by surface relaxation.[31]

The ratio of observed (surface) relaxation times is defined by the spectral density function *J*(*ω*_0_) as:[35]


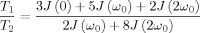
(6)

At high field, this ratio is known to be insensitive to Larmor frequency.[27] Based on typical measured values of *τ*_m,s_ for liquids on an alumina surface,[27] and given that *ω*_0_ is large, we assume that (*τ*_m_/*τ*_s_)^2^≪(*ω*_0_*τ*_m_)^2^≪1. Through a series of trivial algebraic steps it can be shown that *T*_1_/*T*_2_ is proportional to −ln(*τ*_s_/*τ*_m_)/ln(*ω*_0_*τ*_m_). Theoretical calculations previously reported by McDonald et al.[29] suggest that *T*_1_/*T*_2_ is sensitive to small changes in ln(*τ*_m_) but insensitive to large changes in ln(*τ*_s_/*τ*_m_) as *τ*_m,s_ are not independent variables. Therefore, for the purpose of establishing a relationship between surface relaxation and adsorption energy it follows from Equation (3) and the reduced form of Equation (6), given *ω*_0_ is constant, that:


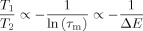
(7)

We define a dimensionless surface interaction parameter *e*_surf_=−*T*_2_/*T*_1_ for the purpose of interpreting surface relaxation as a surface energy.

For the materials of interest in this study, we are concerned only with the dynamics of the adsorbed liquid that give rise to altered NMR relaxation times. However, it is important to consider the extent to which other properties of the sample will influence the observed relaxation times, and the *T*_1_/*T*_2_ ratio. The most important of these are 1) the presence of paramagnetic species on the pore surface, and 2) the presence of magnetic susceptibility contrast between the solid and liquid. First, considering the case of presence of paramagnetic species on the pore surface; such species will alter the sensitivity of the relaxation mechanism to *τ*_m_ and *τ*_s_.[32] Empirical evidence suggests the ratio *T*_1_/*T*_2_ remains independent of the density of surface paramagnetic species (such as Fe^3+^) when the concentration of the paramagnetic impurity is less than 5000 ppm,[36, 37] even though the individual values of *T*_1_ and *T*_2_ are affected by impurities at concentrations greater than 200 ppm.[37] At paramagnetic impurity contents >5000 ppm, re-calibration of the correlation between *e*_surf_ (NMR) and *E*_max_ (TPD) will be necessary. Now considering the second case, the presence of magnetic susceptibility contrast between the solid and liquid will distort the magnetic field such that the diffusion-sensitive *T*_2_ measurement is no longer determined by surface adsorption.[38] We have shown recently that the correct *T*_2_ relaxation time can be extracted in the presence of pore-scale magnetic field inhomogeneities. Neither of these conditions apply to the materials studied here, so the observed *T*_1_/*T*_2_ ratios can be interpreted directly in terms of a surface adsorption energy.

## Experimental Section

### Materials

Zirconia (ZrO_2_), θ-alumina (θ-Al_2_O_3_), γ-alumina (γ-Al_2_O_3_) and silica (SiO_2_) were supplied by Johnson Matthey PLC; anatase titania (TiO_2_-a) and rutile titania (TiO_2_-r) were supplied by Evonik-Degussa. All materials were used as-received in the form of 3–4 mm diameter cylindrical and 4–5 mm length extrudate pellets. Samples for NMR relaxation and TPD measurements were prepared by impregnating the different porous materials in deionised water for at least 24 h. Excess surface water was removed from the pellets by gently contacting them on a pre-soaked filter paper.

The suppliers of the alumina, silica and zirconia samples do not report any measureable paramagnetic impurities. The titania samples do contain some Fe^3+^ species (<100 ppm). BJH analysis of nitrogen adsorption measurements was also performed for all materials studied. The average pore diameter determined was as follows: TiO_2_-a (23 nm); TiO_2_-r (38 nm); γ-Al_2_O_3_ (15 nm); θ-Al_2_O_3_ (7 nm); SiO_2_ (5 nm) and ZrO_2_ (9 nm).

### NMR experiments and data analysis

**Figure 1:**
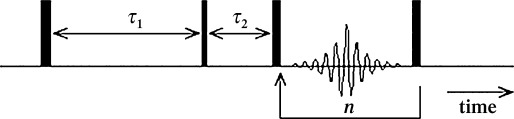
The *T*_1_–*T*_2_ pulse sequence, showing the rf pulses as thin (90°) and thick (180°) vertical bars. The amplitude of each of the *n* spin echoes is recorded as a single datum. The echo centres are separated in time by *t*_e_=2*τ*_2_.

NMR experiments were carried out on a Bruker Biospec (Horizontal Bore) AV 85 MHz spectrometer. Samples were prepared by soaking the different solid materials in the liquid for at least 24 h; the pellets of solid material were dried on a pre-soaked filter paper, in order to remove any excess liquid on the external surface, and finally transferred into 20 mm diameter glass vials, which were then sealed. A standard *T*_1_–*T*_2_ pulse sequence[39] was used to acquire 2D correlation data. The specific sequence of rf pulses is illustrated in Figure 1. A 180° rf inversion pulse rotates the spin magnetisation, initially at equilibrium along the *z* axis, onto the −*z* axis. Longitudinal *T*_1_ relaxation then occurs for a time *τ*_1_; during this time the spin system recovers along the *z* axis and is aligned with the static magnetic field of magnitude *B*_0_. After this recovery time, a 90° rf excitation pulse is applied and rotates the recovered spin magnetisation into the *xy* plane. A series of 180° rf refocusing pulses are then applied to generate a train of *n* spin echoes, each separated in time by *t*_e_=2*τ*_2_. The amplitude of each echo is recorded as a single point, and all echo amplitudes are recorded in a single scan (no chemical resolution). The amplitude of the initial echo is determined by the degree of recovery during *τ*_1_; the envelope of the echo train is described by the transverse *T*_2_ relaxation of the spin ensemble. By repeating the experiment for different *τ*_1_ recovery times, a 2D data matrix is constructed. Here, 16 *T*_1_ recovery delays were used, ranging from *τ*_1_=1 ms to 10 s; *n*=1024 spin echoes were acquired in a single shot with an echo time spacing *t*_e_=0.8 ms. A recycle delay of 10 s was included between each scan to ensure maximum signal was obtained at all times. The total experiment duration was 1.5 h, and included 32 repeat scans to accommodate the rf phase cycle and provide signal averaging to improve the signal-to-noise ratio (SNR) of the data. Elsewhere we have shown that *T*_1_–*T*_2_ data may be acquired in 3 min.[40]

The 2D data are inverted numerically to form a 2D distribution of *T*_2_ correlated against *T*_1_. The NMR data are described by the first kind Fredholm integral equation:


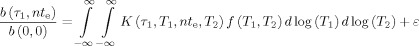
(8)

in which the NMR signal is *b*, *ε* is the experimental error (noise), *f*(*T*_1_,*T*_2_) is the required 2D correlation, and the kernel function *K*(*τ*_1_,*T*_1_,*nt*_e_,*T*_2_) represents the expected form of the data so that:


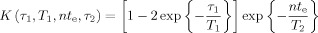
(9)

in which the first exponent describes the *T*_1_ relaxation and the second exponent describes the *T*_2_ relaxation. As the two exponents do not share a common time base, the kernel function is separable, that is, the expected behaviour of the signal amplitude for a given set of (*τ*_1_,*T*_1_) can be determined separately from the expected behaviour of the signal amplitude for a given set of (*nt*_e_,*T*_2_). This separation allows us to solve the Fredholm integral equation in (9) efficiently in vector–matrix form following the method described by Venkataramanan et al.[41] The result is biased to be positive, smooth, and bounded by pre-determined limits in *T*_1_ and *T*_2_. A stable distribution, obtained in the presence of noise, is found using Tikhonov regularisation with the smoothing parameter chosen using the Generalised Cross Validation method;[42] a review of data inversion techniques is presented elsewhere.[43] The inversion is highly susceptible to noise fluctuations and the degree of smoothing increases as the SNR decreases. Variations in distribution shape of less than an order of magnitude on the relaxation time axes are therefore considered to be determined by the signal quality and not the sample.

### TPD experiments

TPD experiments were performed on a CATLAB-PCS (Hiden Analytical), comprising a microreactor module with integrated mass spectrometer. Pellets saturated with water were placed into the glass microreactor in a high purity helium flow at a constant rate of 40 mL min^−1^ and left for one hour at 45 °C until all the physisorbed water was removed. TPD curves of water (*m*=18 amu) were recorded in the range 45–1000 °C with a heating rate of 20 °C min^−1^. Each TPD measurement lasted 3 h. The amount of water desorbed from each porous material during the TPD experiments was calculated by using a calibration factor, which was obtained by measuring the area under the TPD profile corresponding to a known amount of desorbed water from a calibration sample. The analysis of the TPD curves was carried out according to the condensation approximation method reported by Barrie in order to obtain the distribution function of the activation energy of desorption.[44]

### Experimental results and discussion

Having derived Equation (7), we now demonstrate that *T*_1_/*T*_2_ for the systems studied can indeed be related to the strongest relaxation sinks present (i.e., the strongest adsorption sites present)—the strength of the strongest adsorption sites being determined by TPD analysis. We determine the strength of water adsorption in six porous oxides (extruded pellets) used as supports and catalysts: TiO_2_-a (anatase), TiO_2_-r (rutile) (supplied by Evonik-Degussa), ZrO_2_, γ-Al_2_O_3_, θ-Al_2_O_3_ and SiO_2_. We chose water as the adsorbate because it is central to aqueous-phase heterogeneous catalytic processes and, in the context of this work, has the additional advantages of not decomposing during thermally driven desorption.[45]

**Figure 2:**
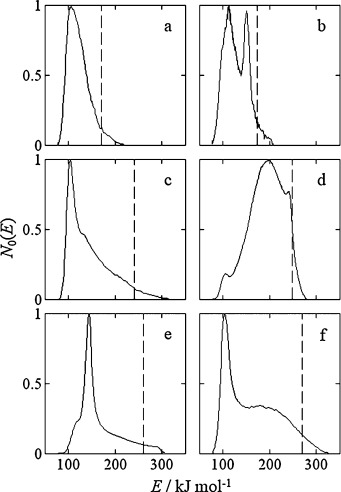
Energy distribution functions obtained from TPD analysis of water in a) TiO_2_-a, b) TiO_2_-r, c) γ-Al_2_O_3_, d) SiO_2_, e) θ-Al_2_O_3_ and f) ZrO_2_. The vertical dashed lines indicate the activation energy of desorption (*E*_max_) at which 95 % of species have desorbed.

The TPD energy distributions for water desorbing from the porous oxides are shown in Figure 2. Due to the uncertainty associated with the tail of the TPD curves, we determine the maximum activation energy of desorption (*E*_max_) where the integral area of the desorption curves is 95 % of the total, see Figure 2.

It is clearly seen that the shape of the TPD curves differs widely between the oxides studied. Ignoring any detailed structure in these plots, we see that the TiO_2_ materials (Figure 2a, b) are associated with significantly lower maximum desorption energies than the other materials studied. Based on the values of *E*_max_ determined from the TPD the strength of the interaction of water with the porous solid decreases in the order:


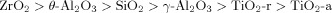
(10)

**Figure 3:**
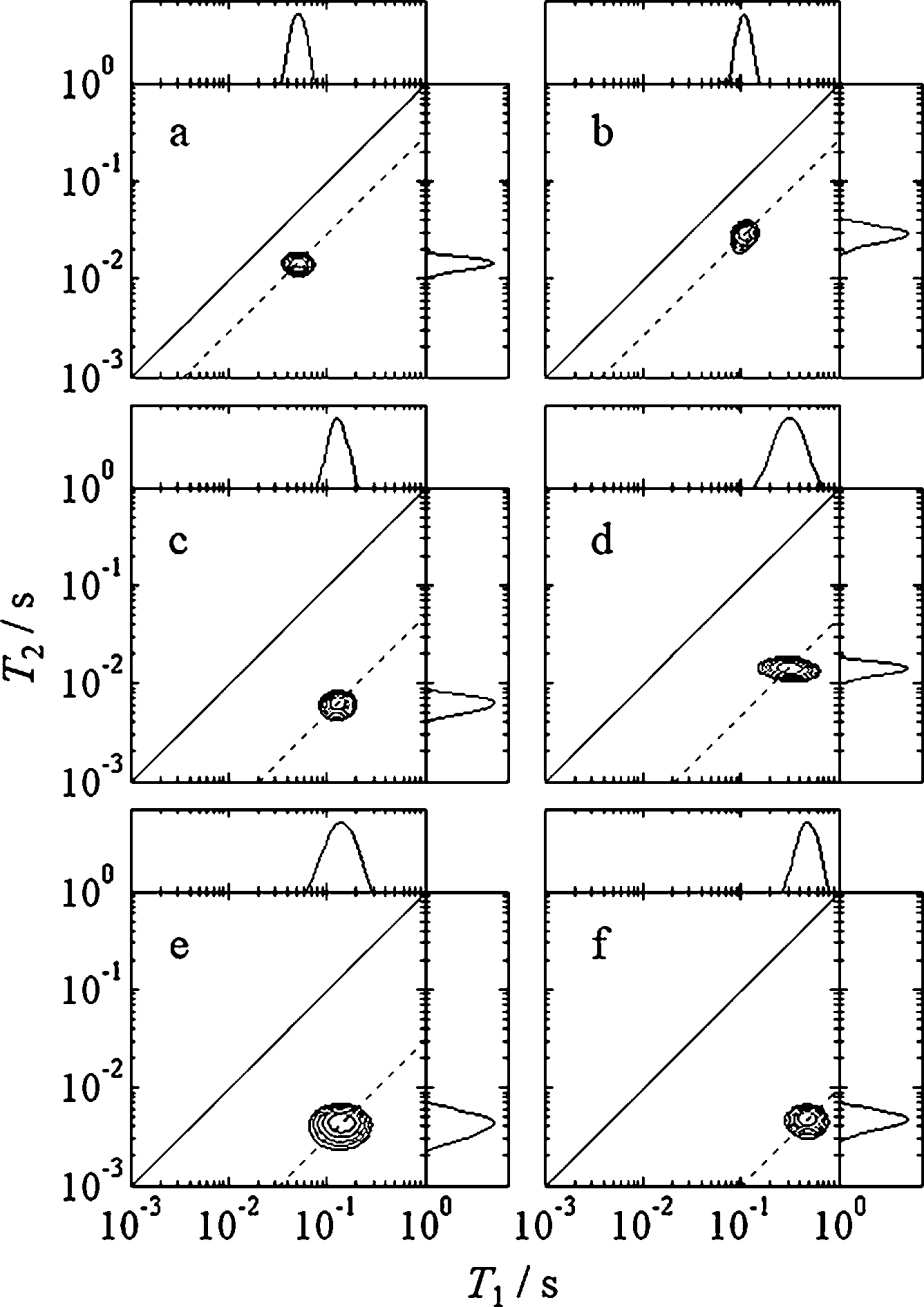
*T*_1_–*T*_2_ correlation plots for water in: a) TiO_2_-a, b) TiO_2_-r, c) *γ*-Al_2_O_3_, d) SiO_2_, e) θ-Al_2_O_3_ and f) ZrO_2_. The solid diagonal line indicates *T*_1_=*T*_2_; the dashed line indicates *T*_1_/*T*_2_ at the maximum of the peak. Projected *T*_1,2_ distributions are shown for clarity.

The *T*_1_–*T*_2_ correlations for water in the porous oxides are shown in Figure 3. In each case, a single relaxation time component is observed; the relaxation times are dominated by surface adsorbed species due to the small pore size and high surface area in these oxides. The ratio *T*_1_/*T*_2_ is obtained from the logarithmic mean of the individual *T*_1,2_ dimensions, which corresponds almost exactly with the maximum intensity of the 2D peak in these mono-modal distributions. In these porous materials with narrow, mono-modal pore size distributions, details of the peak shape are determined predominantly by the raw data quality (degree of smoothing on inversion[43]) and are not considered representative of physical sample properties. The 2D correlations provide a straightforward visual comparison and are required to interpret relaxation results obtained from complicated systems with multiple liquids or diffusive exchange.[24, 29]

**Figure 4:**
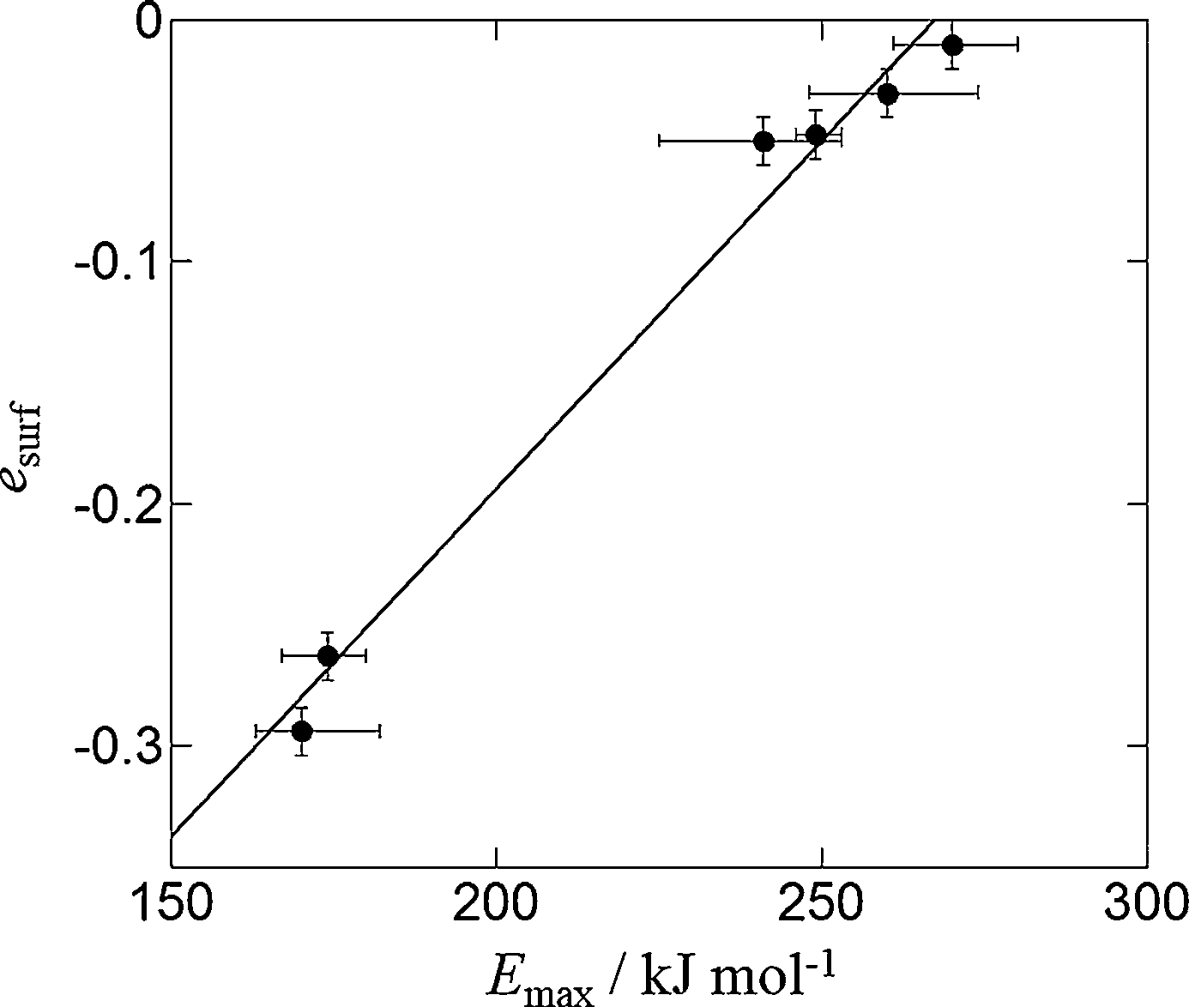
Comparison of *e*_surf_ (NMR) against *E*_max_ (TPD) for water on oxides. A linear relationship (diagonal line) is observed as expected. Symbols correspond to (left to right) TiO_2_-a, TiO_2_-r, γ-Al_2_O_3_, SiO_2_, θ-Al_2_O_3_ and ZrO_2_. Error bars represent the uncertainty in determining the peak position in the *T*_1_–*T*_2_ distributions (*e*_surf_) and ±5 % integral area under the TPD curves (*E*_max_).

A comparison between the NMR relaxation analysis and TPD is achieved by converting the *T*_1_/*T*_2_ ratio into an effective surface interaction parameter, *e*_surf_, using Equation (7). The comparison between *E*_max_ (TPD) and *e*_surf_ (NMR) is given in Figure 4, in which an excellent correlation is obtained between the two measurements. Notably, the TiO_2_ samples have a markedly lower *e*_surf_ value compared to the other oxides, consistent with their TPD spectra being significantly different to those of the other oxides. Elsewhere, a comparison between ZrO_2_ and TiO_2_ surfaces has been carried out by Ignatchenko and co-workers, who concluded, through isotopic exchange experiments and density functional theory (DFT) calculations, that ZrO_2_ has a greater affinity for water than TiO_2_.[46] Our results agree with these previous findings, and the high value of *e*_surf_ obtained for water on ZrO_2_ correlates with the known strong affinity of water for this oxide surface. Measurements of isosteric heat of adsorption have also been used to study porous oxides. Values of the isosteric heat of adsorption for water on SiO_2_[47, 48] have been reported to be comparable to those measured over *γ*-Al_2_O_3_.[49] Again, this is in line with the finding of this work, which shows very similar *e*_surf_ values for SiO_2_ and *γ*-Al_2_O_3_, with SiO_2_ showing a slightly higher value. Overall, our results show that *e*_surf_ for a given liquid adsorbed within a range of chemically similar materials does correlate directly with the maximum activation energy of desorption obtained by TPD analysis, as shown in Figure 4. It follows that if TPD analysis for at least two of the liquid–solid systems under investigation can be obtained, the *e*_surf_ values can be converted to an estimate of the absolute maximum desorption energies characteristic of that system.

The adsorption site densities, calculated from the area under the TPD curve according to the procedure described in the experimental section, were: 0.11 (TiO_2_-a and TiO_2_-r), 0.84 (γ-Al_2_O_3_), 1.38 (SiO_2_), 2.95 (θ-Al_2_O_3_), and 0.60 mmol m^−2^ (ZrO_2_). No strong correlation is found between these values and the *T*_1_/*T*_2_ ratios, as expected from the earlier discussion[29, 30] regarding the relatively low sensitivity of *T*_1_/*T*_2_ to site density compared to the changing nature of interactions between liquids and surfaces of different chemistry.

## Conclusion

In this work we have presented a theoretical analysis to show that the ratio of NMR relaxation times, *T*_1_/*T*_2_, can be related directly to the adsorbate–adsorbent interaction energy characterising adsorption in a porous material, and we introduce a quantitative metric *e*_surf_ (based on the relaxation time ratio) characterising the strength of this surface interaction. We confirm that the *T*_1_/*T*_2_ ratio is insensitive to pore geometry, allowing adsorption of liquids to be compared between different materials. Given that the relaxation time characteristics of the nuclear spin system will be dominated by the strongest adsorption sites, the hypothesis that −*T*_2_/*T*_1_ should correlate directly with the maximum activation energy of desorption observed in a temperature-programmed desorption experiment was tested; the data were found to support this hypothesis. Given this result, we now have a physical interpretation of the NMR relaxation data in terms of a surface activation energy of desorption. It follows from the analysis that for a series of materials for which at least two calibration values are obtainable by TPD, the *e*_surf_ parameter yields a direct estimate of the maximum activation energy of desorption from the surface. Although the method has been demonstrated for water, the NMR relaxation analysis can be extended to probe non-invasively any adsorbed species, notably organic molecules. Further, given that NMR relaxation is readily extended to measurements at elevated conditions of temperature and pressure, these results suggest that the technique might be considered a useful addition to the toolkit of techniques employed to characterise adsorption in porous media used for applications in catalysis and separations processes. Beyond these applications, demonstrating that *T*_1_/*T*_2_, when expressed as *e*_surf_, is a quantitative measure of interaction strength has implications for determining surface wettability in oil-field reservoir rocks, an important parameter for predicting oil recovery. An improved correlation between relaxation time and surface adsorption energy could be obtained by measuring *T*_1_/*T*_2_ as a function of temperature or field strength, although such procedures for providing measurements of surface interaction strength would be considerably more time consuming.

NMR relaxation has been validated as a robust and versatile tool for quantitatively comparing liquid–solid interactions in porous materials. In principle, the method is applicable to the characterisation of both liquid and gas phase adsorption, although signal-to-noise considerations may limit its applicability to gas–solid interactions. This limitation and the consideration of chemically different surfaces (e.g., carbon-based), or materials with much smaller pore size (e.g., zeolites) are the subject of further consideration.
